# The association of maternal smoking and other sociobehavioral factors with dental caries in toddlers: A cross-sectional study

**DOI:** 10.3389/fped.2023.1115978

**Published:** 2023-04-03

**Authors:** Piotr Sobiech, Dorota Olczak-Kowalczyk, Karolina Spodzieja, Dariusz Gozdowski

**Affiliations:** ^1^ Department of Paediatric Dentistry, Medical University of Warsaw, Warszawa, Poland; ^2^ Department of Experimental Design and Bioinformatics, Warsaw University of Life Sciences, Warsaw, Poland; ^3^ Department of Agriculture and Biology, Warsaw University of Life Sciences, Warsaw, Poland

**Keywords:** early childhood caries, maternal smoking, socio-demographic factors, toddlers, caries in children

## Abstract

**Background:**

Severe early childhood caries (S-ECC) is a form of dental caries in toddlers, which can strongly affect general health and quality of life. Studies on factors that can contribute to the development of caries immediately after tooth eruption are sparse. The aim of this study was to assess the role of sociobehavioural factors and pre- and postnatal exposure to tobacco smoke in the aetiology of dental caries in children up to 3 years old.

**Methods:**

A cross-sectional study was conducted between 2011 and 2017 to assess oral health and teething in urban children 0–4 years of age. The number of teeth and surfaces with white spot lesions (d_1,2_), as well as decayed (d), missing (m), and filled (f) teeth classified according to ICDAS II was evaluated in a dental office setting. d_1,2_dmft and d_1,2_dmfs were calculated. Severe early childhood caries was diagnosed for d_1,2_dmfs > 0. Parents completed a self-administered questionnaire on socioeconomic factors, maternal health, course of pregnancy, child's perinatal parameters, hygiene and dietary practices, as well as maternal smoking during and after pregnancy. Data on children aged 12–36 months were collected and analysed statistically using the *t*-test, Spearman rank correlations and Poisson regression. Significance level was set at 0.05.

**Results:**

Dental caries was found in 46% of 496 children aged 12–36 months. Mean d_1,2_dmft and d_1,2_dmfs were 2.62 ± 3.88 and 4.46 ± 8.42, respectively. Tobacco smoking during and after pregnancy was reported by 8.9% and 24.8% of women, respectively. Spearman's rank correlation analysis confirmed a relationship between S-ECC and parental education, maternal smoking, bottle feeding, avoiding springy foods, number of meals, and the age of tooth brushing initiation. Pre- and postnatal exposure to tobacco smoke increased the risk of S-ECC especially in children in age 19–24 months. Maternal smoking was correlated with the level of education and dietary practices.

**Conclusion:**

Our study confirmed that prenatal smoking is associated with increased risk of severe-early childhood caries (S-ECC) while the association with post-natal smoking is also evident, the increase in risk is not statistically clear. Both maternal smoking and the child's tooth decay are associated with poor parental education and other improper oral health behaviours. The positive impact of quitting smoking on the oral health in children should be part of anti-smoking advice.

## Background

Early childhood caries (ECC), defined as the presence of one or more decayed, missing, or filled primary teeth in children aged 71 months or younger, remains a serious global health issue (1–3). Dental caries in infants and the youngest children, i.e., caries onset shortly after eruption, poses a particular threat. Low enamel and dentin mineralisation in newly erupted teeth promotes high dynamics of carious processes, which may cause crown destruction, pulpal complications and periodontal infections in a short time. Therefore, according to the AAPD definition, in children younger than 3 years of age, any sign of smooth-surface or occlusal caries is indicative of severe early childhood caries (S-ECC) (1).

The incidence of S-ECC in developed countries is estimated at 1%–12.6% among 2-year-olds (4, 5) and 46% among children aged 25–36 months (6). Research conducted for many years identified the major causative factors of ECC, such as dietary behaviours involving exposure to carbohydrates contributing to the development of cariogenic biofilm. The role of demographic (e.g., socio-economic and cultural characteristics) and medical factors (low birth weight) is also emphasised (2, 3, 7–9). The relationship between ECC and parental behaviours, including maternal prenatal smoking and child's exposure to tobacco smoke, is increasingly emphasised (10).

Analyses of the population of pregnant women in Poland proved that over 18% of women smoked cigarettes during the last 3 months preceding pregnancy, 6.8% smoked at least one cigarette in first month of pregnancy, 7.7% smoked in the last trimester, 3.6% of women smoked after birth (11).

There is a growing amount of prospective studies on early childhood caries starting with prenatal recruitment. It was concluded that maternal vitamin D levels may affect tooth calcification, predisposing to early childhood caries (12). Another study investigated whether a relation exists between levels of vitamin D in the umblical cord and caries in children (13). No correlation has been found between perinatal stress and development of early childhood caries in children (14). Recent metaanalysis showed reduced caries incidence in children whose mothers received prenatal oral health (15).

Several studies have been conducted among children <3 years of age (4–9). Considering the consequences of carious lesions in the youngest children, it seems necessary to expand knowledge on this issue.

The aim of this paper was to identify whether pre- and postnatal exposure to tobacco smoke can be associated with increased risk of S-ECC in children.

## Methods

This cross-sectional study involved clinical examination in children 12–36 months of age and a questionnaire among their parents/legal guardians. The study was conducted as part of a 2011–2018 programme assessing oral health and teething in children ≤3 years of age. Children were recruited by placing information in the press on motherhood, as well as in nurseries and paediatric clinics. The study was approved by the Bioethics Committee of the Medical University of Warsaw (No. KB/221/2009). A written consent of a parent/legal guardian to participate in the study, residence in Warsaw (drinking water fluoride levels <0.3 mg/L), child's cooperation allowing for clinical examination, and the presence of at least four erupted maxillary incisors were inclusion criteria. A child's history of chronic disease or pharmacotherapy likely to affect oral health (e.g., anti-allergic treatment), lack of cooperation, completed questionnaire were exclusion criteria. The questionnaire included questions on socioeconomic factors (parental education and age, number of children in the family, economic status, smoking), medical factors (such as current maternal chronic diseases, the course of pregnancy and child's perinatal parameters, the use of endogenous fluoride prevention, child's dietary habits (breast/bottle feeding and the age of its termination, the daily number of meals, preferences related to springy foods, and the presence of sugar in food products consumed by the child), toothbrushing and age of toothbrushing initiation, and the type of toothpaste used. The questionnaire was checked and verified after two pilot studies in 20 parents reporting their children for a routine check-up.

Clinical examination was performed in a dental office setting to assess oral health status, including the number of erupted teeth, the presence of white spot lesions, filled and missing teeth, enamel opacities and hypoplasia, as well as fistula and abscess. All dental surfaces in subsequent quadrants were examined. We assessed carious lesions in accordance with the International Caries Detection and Assessment System (ICDAS) (16) (Table 1). We calculated d_1,2_dmfs and d_1,2_dmft, where d_1,2_ stands for ICDAS II codes 1 and 2, and p stands for at least code 3. We determined the incidence of S-ECC, expressed as the percentage of children with d_1,2_dmfs > 0 (1). Examinations were performed by three trained and calibrated dentists specialised in paediatric dentistry. Kappa Cohen coefficient of 0.89 to 0.95 was obtained.

**Table 1:** ICDAS II codes and criteria used in the diagnosis of coronary caries.

Code	Description
1	Sound tooth surface: No evidence of caries after 5 s air drying
2	First visual change in enamel: Opacity or discoloration (white or brown) is visible at the entrance to the pit or fissure seen after prolonged air drying
3	Distinct visual change in enamel visible when wet, lesion must be visible when dry Localized enamel breakdown (without clinical visual signs of dentinal involvement) seen when wet and after prolonged drying
4	Underlying dark shadow from dentine
5	Distinct cavity with visible dentine
6	Extensive (more than half the surface) distinct cavity with visible dentine

The results were analysed for the total group of children and for four age subgroups: 12–18, 19–24, 25–30, and 31–36 months.

The results were presented as means and standard deviations or the number of observations and percentages. Single and multiple (with covariates) Poisson regression was used in statistical analysis to evaluate the relationships between S-ECC and maternal smoking during and after pregnancy. The results of Poisson regression were presented as regression coefficients together with *p*-values. Spearman's rank correlations were used for the evaluation of relationships between medical and socio-behavioural factors and S-ECC. Statistical comparisons of means were conducted using *t*-test. Statistica 13 and R 3.5 software were used for the analyses. Significance level for all analyses was set at 0.05.

## Results

From 907 children reported for the participation in the programme for the assessment of oral health and teething, 496 were included in the study. Age below 12 months or over 36 months and incorrectly completed questionnaires were the causes of exclusion. All questionnaires were completed by mothers of the enrolled children.

Socioeconomic characteristics of parents/legal guardians and children is summarised in Table 2. The number of children per family was 1 to 4 (mean 1.63 ± 0.71). A total of 126 women reported having a chronic disease. Allergies (*n* = 47), hormonal disorders (*n* = 31), diabetes (*n* = 17), hypertension (*n* = 11), and epilepsy (*n* = 6) were most common. Pregnancy-related health issues were confirmed by 120 women. These included infections (*n* = 30), anaemia (*n* = 31), diabetes (*n* = 28), hypertension (*n* = 21). Epilepsy (*n* = 6) and hormonal disorders (*n* = 4) were less common. A total of 123 mothers (24.8%) were smokers; 44 (8.9%) mothers smoked during pregnancy.

**Table 2:** Socioeconomic characteristic of parents.

Parameters	Number *N* (%)
**Maternal age**
≤25 years	43 (8.7%)
26–35 years	364 (73.4%)
≥36 years	89 (17.9%)
**Paternal age**
≤25 years	21 (4.2%)
26–35 years	329 (66.3%)
≥36 years	146 (29.5%)
**Maternal education**
Primary	19 (3.8%)
Secondary	157 (31.7%)
Higher	320 (64.5%)
**Paternal education**
Primary	16 (3.2%)
Secondary	156 (31.5%)
Higher	324 (65.3%)
**Maternal self-assessment of economic status**
Low	8 (1.6%)
Average	234 (47.2%)
High	254 (51.3%)

The mean age of children in the study group was 24.16 ± 6.92 months. The size of and the mean age in age subgroups, as well as data on pregnancy duration and mode of its termination, perinatal parameters, child feeding manner, and oral hygiene in the child, as declared by mothers, are shown in Table 3. All mothers confirmed sugar intake in their children. A total of 47 (9.5%) parents did not brush their child's teeth at the time of the study, and 79 (15.9%) parents began to brush their children's teeth at the age of 2 years. In the group of children with brushed teeth, the minimum and the maximum age of tooth brushing initiation were 4 and 28 months, respectively. None of the children received fluoride tablets or drops.

**Table 3:** Age groups of children, perinatal parameters, mode of feeding, and oral hygiene procedures in the child.

	*N* (%)	Mean ± SD
**Child's age**
12–18 months	135 (27.2%)	15.52 ± 2.06 months
19–24 months	128 (25.8%)	21.76 ± 1.83 months
25–30 months	129 (26.0%)	27.85 ± 1.77 months
31–36 months	104 (21.0%)	33.75 ± 1.55 months
**Pregnancy and perinatal parameters**
Single pregnancy	463 (93.3%)	-
Vaginal delivery	333 (67.1%)	-
Pregnancy duration	-	38.88 ± 2.29 weeks
<28 weeks	5 (1.0%)	
Birth weight	-	3366.44 ± 561.59 g
<2,500 g	32 (6.5%)	1998.94 ± 563.66 g
**Perinatal complications**
Ischaemia	28 (5.6%)	-
Infection treated with antibiotic	19 (3.8%)	-
**Child feeding**
The child was not breastfed	116 (23.4%)	-
The child was not bottle fed	174 (35.1%)	-
Breastfeeding >12 months	127 (25.6%)	-
Bottle feeding >12 months	112 (22.6%)	-
Number of meals per day	-	5.65 ± 1.07
>5/day	228 (46.0%)	-
Avoiding springy foods	105 (21.2%)	-
**Toothbrushing in the child**	449 (90.5%)	-
**Age of toothbrushing initiation**	-	10.67 ± 4.31
**A history of dental visit**	249 (50.2%)	-

The incidence of S-ECC in the total group was 46%. The incidence of caries, as well as the number of carious teeth and surfaces increased with children's age (Table 4). No statistically significant relationships between oral health and child's gender were found.

**Table 4:** The number of assessed teeth, the incidence and severity of S-ECC in the total group and subgroups.

Age	Number of teeth	d_1,2_dmft	d_1,2_dmfs	d_1,2_dmfs > 0
	mean ± SD			*n* (%)
12–18 months	9.21 ± 3.67	0.71 ± 1.67	1.09 ± 3.07	20/135 (14.8%)
19–24 months	14.27 ± 2.83	2.05 ± 2.91	3.80 ± 6.61	56/128 (43.7%)
25–30 months	17.48 ± 2.73	3.32 ± 4.28	5.02 ± 7.72	75/129 (58.1%)
31–36 months	19.40 ± 1.28	4.02 ± 4.77	7.27 ± 11.98	77/104 (72.0%)
Total	15.40 ± 4.60	2.62 ± 3.88	4.46 ± 8.42	228/496 (46.0%)

Spearman's correlation analysis showed no significant impact of medical factors, the course or manner of termination of pregnancy, or child's perinatal parameters on the severity of ECC. However, it showed the importance of socioeconomic factors, parental education in particular, and health behaviours (Table 5). An additional analysis showed statistically significant relationships between maternal smoking and child feeding manner (*r* = 0.170 for exclusive bottle feeding) and the age of toothbrushing initiation (*r* = 0.108). Also, statistically significant relationships were found between socioeconomic factors, parental education in particular, and maternal smoking (*r* = −0.303 and −0.332, respectively), including the period of pregnancy (*r* = −0.293 and −0.257, respectively). Spearman's coefficients for parental age and family economic status were lower than 0.100.

**Table 5:** Spearman's correlation coefficients showing the relationships between the prevalence and severity of S-ECC and socioeconomic factors and health behaviours related to the child.

Parameters	d_1,2_dmfs				
	Total group	Age (months)			
		12–18	18–24	25–30	31–36
**Maternal education**	**−0.285** *	**−0.203** *	**−0.293** *	**−0.358** *	**−0.340** *
**Paternal education**	**−0.289** *	**−0.254** *	**−0.323** *	**−0.374** *	**−0.260** *
Maternal age	−0.138*	−0.148	−0.238*	−0.046	−0.171
Paternal age	−0.144*	−0.191	−0.154	−0.152	−0.149
Self-assessed economic status	−0.177*	0.023	−0.217*	−0.123	−0.128
**Current maternal smoking**	**0.212** *	0.114	**0.282** *	**0.299** *	0.090
Maternal smoking in pregnancy	0.188*	0.077	0.090	0.448*	−0.060
Exclusive bottle feeding (the child has never been breastfed)	0.164*	0.266*	0.213*	0.205*	0.094
**Number of meals consumed by the child per day**	**0.263** *	0.079	0.161	**0.378** *	**0.369** *
Avoiding springy foods	0.106*	0.064	0.236*	0.170	−0.034
Age of toothbrushing initiation	0.148*	0.071	0.086	0.203*	0.127

* Statistical significance level *p* < 0.05.

Significantly higher d_1,2_dmfs was found in the children whose mothers smoked during pregnancy and postnatally, which was most significant for children in age 19–24 months (Table 6). Results of simple and multiple Poisson regression confirmed the contribution of these unhealthy behaviours to the prevalence of caries in children because most of the regression coefficient's were positive and many of them significant (*p* < 0.05).

**Table 6:** The prevalence and severity of caries in children whose mothers smoked vs. did not smoke during pregnancy and postnatally; Regression coefficients and adjusted regression coefficients (confounders: education, age, economic status, exclusive bottle feeding, number of meals, avoiding springy foods, and the age of tooth brushing initiation) based on Poisson regression model where dependent variable was d_1,2_dmfs.

	Prevalence of ECC (d_1,2_dmfs >0)	Regression coefficient (*p*-value)	Adjusted regression coefficient (*p*-value)	d_1,2_dmfs	*p*
**12**–**18 months**					
Maternal smoking during pregnancy and postnatally					
Yes	1/3 (33.3%)	0.64 (0.129)	0.14 (0.751)	2.00 ± 3.46	0.603
No	19/102 (18.6%)			1.06 ± 3.07	
Maternal smoking postnatally, but not during pregnancy					
Yes	5/19 (26.3%)	0.24 (0.289)	0.22 (0.371)	1.32 ± 2.58	0.720
No	15/86 (17.4%)			1.03 ± 3.18	
Maternal smoking during pregnancy and/or postnatally					
Yes	6/22 (27.3%)	0.34 (0.103)	0.22 (0.330)	1.41 ± 2.63	0.581
No	14/83 (16.9%)			1.00 ± 3.19	
**19–24 months**					
Maternal smoking during pregnancy and postnatally					
Yes	4/7 (57.1%)	0.64 (<0.001)	0.46 (0.006)	6.86 ± 8.97	0.201
No	52/122 (42.6%)			3.62 ± 6.46	
Maternal smoking postnatally, but not during pregnancy					
Yes	13/18 (72.2%)	0.97 (<0.001)	0.35 (0.002)	3.09 ± 10.07	0.002
No	43/111 (38.7%)			8.17 ± 5.62	
Maternal smoking during pregnancy and/or postnatally					
Yes	17/25 (60.2%)	1.01 (<0.001)	0.52 (<0.001)	7.80 ± 9.60	<0.001
No	39/104 (37.5%)			2.84 ± 5.30	
**25**–**30 months**					
Maternal smoking during pregnancy and postnatally					
Yes	18/19 (94.7%)	1.30 (<0.001)	0.39 (<0.001)	13.37 ± 11.77	<0.001
No	57/114 (50.0%)			3.63 ± 5.83	
Maternal smoking postnatally, but not during pregnancy					
Yes	10/19 (52.6%)	−0.21 (0.070)	0.02 (0.854)	4.16 ± 5.04	0.056
No	65/114 (57.0%)			5.17 ± 8.09	
Maternal smoking during pregnancy and/or postnatally					
Yes	28/38 (60.2%)	0.91 (<0.001)	0.29 (0.002)	8.76 ± 10.08	<0.001
No	47/95 (49.5%)			3.53 ± 5.99	
**31–36 months**					
Maternal smoking during pregnancy and postnatally					
Yes	7/15 (46.7%)	0.19 (0.043)	−0.07 (0.500)	8.60 ± 14.86	0.649
No	70/114 (61.4%)			7.10 ± 11.61	
Maternal smoking postnatally, but not during pregnancy					
Yes	16/23 (69.6%)	0.50 (<0.001)	−0.06 (0.508)	10.78 ± 5.04	0.121
No	61/106 (57.5%)			6.51 ± 8.09	
Maternal smoking during pregnancy and/or postnatally					
Yes	23/38 (60.2%)	0.48 (<0.001)	−0.08 (0.310)	9.92 ± 14.83	0.105
No	54/91 (59.3%)			6.16 ± 10.45	

*Statistical significance level *p* < 0.05.

## Discussion

Our results confirm that S-ECC is a common problem in Poland. Epidemiological studies conducted in Poland in 2017 estimated the incidence of ECC in 3-year-olds, diagnosed only as the presence of cavitated caries, at 41.1%, while the same incidence was 52% in the cities of Mazowieckie Province (17). Slightly higher incidence of S-ECC in our group of toddlers is certainly due to the definition of caries, which encompassed both cavitated and non-cavitated lesions.

Despite the lack of long-term follow-up, which is a limitation of cross-sectional study design, the obtained data revealed factors related to the development of caries immediately after eruption. Our study confirmed the major role of the manner of child feeding, socioeconomic factors, and maternal smoking, both during pregnancy and after birth. Important factor associated with smoking in Poland is age and level of education. It was found that among heavy smokers are mothers with basic education level while people with higher education are the least likely to smoke. We did not collect data on the daily frequency of consuming sugar-containing products by the children as the most discordant answers during questionnaire preparation were obtained for the question on the frequency of consuming these products. Additionally, interviews with parents revealed their insufficient knowledge on the content of sugars fermentable by bacteria in the products consumed by their children, such as fruit yoghurts. Therefore, we only enquired whether extra sugar was added to the products consumed by the child, this increases the chances of recall bias All children in the study group consumed such products. The 2017 Polish epidemiological studies showed that up to 86.7% of parents of 3-year-olds added sugar in the meals for their children during their first 2 years of life, which corresponds to our findings (17). The group differed in terms of eating habits. Spearman's correlation analysis confirmed adverse effects of exclusive bottle feeding, i.e., giving up breastfeeding during infancy, and frequent consumption of meals by the child, which corresponds to the findings presented by other authors (2, 3, 6–9, 18). It is worth emphasising that bottle feeding was more important in younger children, whereas the number of meals—in older children. Avoiding springy foods was found to be an important aetiological factor for ECC among children aged 18–30 months. It is a known fact that the chewing function is an important factor stimulating salivary flow, acid neutralisation and self-cleaning of the oral cavity. There was also a relationship (in the total study group) between ECC and age at which toothbrushing was initiated. Considering age groups, the impact of this factor was clearly visible only in the group of 25–30-month-olds. There is a large body of evidence in literature for the relationship between oral hygiene behaviours and the incidence of ECC (8, 9, 19, 20). Starting to brush at an earlier age and parent-assisted brushing can often reduce the risk of caries, which is consistent with the results of some studies (21, 22).

Socioeconomic factors are considered to be strongly associated with S-ECC. The relationship between ECC and socioeconomic status (SES) is well-documented. ECC is usually found in children from families with low economic status and poor parental education (2, 3, 23–25). It is believed that malnutrition or pre- and perinatal malnutrition, insufficient exposure to fluoride and higher preference for sweet products are the causes (26–30). Our study showed no relationship between ECC and medical or perinatal factors, including low births weight. There were also weak correlations between self-assessed economic status and parental age. This may result from respondents’ subjective assessment, as well as limited diversity of the group in terms of economic status. Warsaw is a part of the Mazowieckie Province, where average household monthly income rate is 120% in relation to the national average (31). It was found that parental education (both maternal and paternal) was a factor most strongly associated with ECC and maternal smoking in pregnancy.

There are ongoing discussions on whether there is a correlation between maternal smoking in pregnancy and caries in the child. A systematic review of literature demonstrated that seven out of eight studies confirmed the relationship between maternal smoking in pregnancy and ECC. One study showed that children whose mothers smoked at least 5 cigarettes per day during pregnancy presented with higher severity of caries compared to children of non-smoking mothers (32). A 2018 meta-analysis demonstrated a relationship between ECC and both smoking in pregnancy [OR = 1.46 at 95% CI 1.41–1.52 (*Z*-test, *p* = 0.000), without heterogeneity (Q = 0.91, *p* = 0.824; *I*^2^ = 0%)], as well as postnatal maternal smoking [OR = 1.72 (95% CI 1.45–2.05) and high heterogeneity (Q = 76.59, *p* = 0.00; *I*^2^ = 83.01%)] (33). A 2019 systemic review and meta—analysis of risk factors for ECC confirmed that parental smoking and smoking during pregnancy is a risk factor for ECC amid high income countries (34). A 2020 systematic review confirmed that both preterm birth and ECC have common variables affecting the prevalence such as parental education and smoking (35). Swedish retrospective cohort study revealed that smoking and BMI are associated with childhood caries (36). In our study, due to the risk of overestimating the strength of correlation using OR, we found prevalence ratios (PRs) and adjusted PR, in which socioeconomic factors and oral behaviour identified in Spearman's analysis were the confounders, to be more appropriate (37). Both of them confirmed that pre- and postnatal exposure to tobacco smoke may be a risk factor for ECC.

Similar cross—sectional studies concerning parental smoking and ECC were performed in Italy and Lithuania. Results revealed that smoking habit of parents was significantly related to ECC since smokers tend to have poor oral habits and low oral health awareness (38, 39).

Several hypotheses have been proposed to explain the relationship between ECC and fetal/child exposure to tobacco smoke. Negative effects of smoking in pregnancy on tooth bud mineralisation (40–42) and a relationship between secondhand smoke (SHS) and early eruption of primary teeth have been shown (43). High maternal levels of cariogenic bacteria are also a known risk factor for ECC. Lindemeyer et al. confirmed higher levels of cariogenic bacteria in smoking vs. non-smoking mothers (44). It was shown that nicotine promotes the attachment of *S. mutans* to dental surface (45). It contributes to higher counts of these bacteria (46) and higher metabolic activity of the biofilm (47).

It is also believed that smoking influences saliva by lowering its buffer capability, altering its chemical agent and bacterial components, and therefore promotes the formation of a caries-susceptible environment (48). Avçar et al. reported a decrease in pH, buffer capacity and salivary flow due to tobacco smoke exposure (49). It is also known that immunosuppression may promote *Streptococcus mutans* colonisation (43).

It is believed that the relationship between smoking in pregnancy and secondhand smoke and caries in children may be due to the overlap of socioeconomic, educational and behavioural factors. Second hand smoking may have a direct impact on teeth and oral environment. Second hand smoke may induce inflammation of oral mucosa, cause salivary gland impairment and cause immune dysfunction. This may lead to aggregation of cariogenic bacteria and development of caries (50). Our Spearman's correlation analysis confirmed correlations between socioeconomic factors and both smoking and ECC. It also showed poorer care for the child's well-being among smoking mothers, which was reflected by delayed introduction of toothbrushing and giving up breastfeeding. It is worth emphasising that the effects of child exposure to tobacco smoke were visible already at the age of 2 years. This corresponds to the findings presented by other authors. Also, a relationship was confirmed between passive child exposure to smoke and low socioeconomic status (51). Furthermore, poorer hygiene behaviours and higher exposure to sugar were observed among children whose parents were smokers (52–54). Attention was also paid to an increased risk of ECC in bottle-fed children from families with low socioeconomic status who were exposed to tobacco smoke (55).

The strength of our study is that data we gathered may be used to explore different variables and can help the researchers to proceed further investigation concerning risk factors for ECC. However, the limitation we faced is fact, that only urban children and their parents were participants in this cross—sectional study. Therefore it is not representative of an entire polish population.

It is noteworthy that we observed differences in the role of different factors in various age groups. The importance of postnatal exposure to tobacco smoke became apparent already at the age of 2 years, which seems to emphasise the role of its direct impact.

At the same time, the introduction of socioeconomic factors and oral behaviours as confounders into the logistic regression model only slightly reduced the value of the prevalence ratio. This confirms not only the relationship between fetal/child exposure to passive smoking and ECC, but also its independence of other factors involved in the aetiology of caries. We did not assess the importance of the degree of exposure to tobacco smoke due to discordant answers on the number of cigarettes per day at the stage of questionnaire development and the inability to measure the nicotine level in saliva. However, other authors noted increased incidence of caries in children exposed to tobacco smoke for more than 30 min compared to children with shorter exposure. Also, a significant positive relationship was shown between blood cotinine levels and the odds ratio for caries presence (56, 57).

## Conclusion

Undoubtedly, prospective studies are needed to provide evidence that there is a relationship between ECC and passive pre- and postnatal exposure to nicotine smoke. Despite methodological limitations, our study confirmed that passive prenatal exposure of children to tobacco smoke and postnatal maternal smoking may be associated with increased risk of S-ECC, regardless of other factors. We also confirmed the impact of parents’ education on their attitude and oral health behaviours, as well as the role of these factors in the aetiology of dental caries. Moreover, we indicated the coexistence of maternal cigarette smoking with other negative health behaviors and the relationship between maternal smoking on the level of education and economic status of parents.

## Data Availability

The raw data supporting the conclusions of this article will be made available by the authors, without undue reservation.
